# Protective Effect of Synbiotic Supplementation Against *Salmonella* Typhimurium Infection in Young Broiler Chickens

**DOI:** 10.3390/ani16091411

**Published:** 2026-05-05

**Authors:** Walter Rivera Pérez, Elías Barquero Calvo, Aida Chaves Hernández, Catalina Salas Duran

**Affiliations:** 1 Programa de Posgrado en Ciencias Agrícolas y Recursos Naturales, Universidad de Costa Rica, San Jose 11501, Costa Rica; 2 Programa de Investigación en Enfermedades Tropicales, Escuela de Medicina Veterinaria, Universidad Nacional, Heredia 40104, Costa Rica; 3 Laboratorio de Patología Aviar, Escuela de Medicina Veterinaria, Universidad Nacional, Heredia 40104, Costa Rica

**Keywords:** immune modulation, cytokine expression, bacterial colonization, gut health

## Abstract

*Salmonella* infection in poultry remains a major concern due to its economic impact and public health risks. Synbiotics have emerged as a natural strategy to improve gut health and resistance to enteric pathogens. This study evaluated the immune response and early protective effects of a synbiotic in broiler chickens using a reduced in vivo model of *Salmonella* Typhimurium infection. A total of 44 broiler chickens were randomly assigned to four dietary treatments: control (CT), control plus synbiotic (CT + Syn), challenged (ST), and challenged plus synbiotic (ST + Syn). Birds were challenged at 7 days of age and euthanized at day 12 for sample collection. Synbiotic supplementation reduced bacterial colonization and adhesion in the ileum, as assessed by intestinal content isolation and immunofluorescence analysis. Additionally, the synbiotics mitigated lesion severity in the intestine, liver, and heart, increased anti-inflammatory IL-10 expression, and reduced pro-inflammatory cytokines IL-1β and TNF-α. Ileal histomorphometry showed increased epithelial height in the ST + Syn birds, while intestinal permeability was not affected. Improved body weight was also observed compared to the challenged birds without supplementation. In conclusion, synbiotic supplementation provided early protection against *S*. Typhimurium infection by modulating immune responses, preserving intestinal morphology, and supporting weight gain in broiler chickens.

## Simple Summary

*Salmonella* infection in chickens is a serious problem because it harms animal health, farm profits, and human food safety. This study evaluated whether adding a synbiotic (a mixture of beneficial bacteria and nutrients) to chicken feed could help protect young chickens from early infection. Chickens that received the synbiotic showed fewer bacteria in their intestines, less tissue damage, less inflammation, and better weight gain. These results suggest that synbiotics may serve as a natural approach to supporting poultry health and potentially reducing foodborne transmission risks.

## 1. Introduction

Recently, poultry research has emphasized the importance of the birds’ gastrointestinal tract when considering its complexity and additional gastrointestinal functions, thereby coining the concept of “intestinal health” [1,2]. A healthy gastrointestinal tract relies on numerous interrelated factors, including diet, digestion and absorption processes, immune function, gastrointestinal barrier integrity, and microbiota stability [2,3].

Avian health is a highly relevant area of research due to the need to understand and promote their well-being and resistance to pathogens [4]. Concerns about the use of antibiotics in feed, particularly because of their potential to generate residues and bacterial resistance, have driven the search for alternative strategies to maintain optimal gut health and growth in birds [5,6]. In this context, functional supplements such as probiotics [7,8], prebiotics [9,10], and synbiotics [11,12] are being investigated as promising tools to strengthen the microbiota, modulate immune responses, and prevent diseases caused by pathogens such as *Salmonella*.

Synbiotics are a synergistic combination of prebiotics and probiotics that optimize host performance [13]. These additives enhance the survival and activity of beneficial microorganisms in the gut, promote the diversity and stability of the microbial community, and foster positive interactions with the gastrointestinal epithelium and the host’s immune system [14]. The use of synbiotics is more effective than individual preparations, and their application during the first hours of life promotes the colonization of the gastrointestinal tract by probiotic strains [1,3].

*Salmonella*, one of the most common zoonotic pathogens, poses a significant threat to both animal and human health globally [6]. The invasion of this bacteria causes various clinical symptoms and intestinal disturbances, resulting in decreased animal performance and substantial economic losses [15]. Greater emphasis should be placed on implementing a multi-intervention approach, as reliance on a single strategy does not reliably achieve effective control [16]. One potential strategy to enhance avian health is the modulation of gut microbiota at an early stage [17]. In this context, synbiotics have been shown to exert a synergistic effect, promoting intestinal microbial balance by selectively stimulating beneficial bacteria while inhibiting pathogenic species [18].

The influence of synbiotics on the intestinal microbiota is considered fundamental to their biological effects, and their benefits have been widely documented in studies on avian health [11,12]. Most studies have evaluated probiotics and prebiotics separately, while only a few investigations have utilized synbiotics [19]. Therefore, the present study aims to evaluate the immune response of broiler chickens and the early protective effect of a synbiotic, including its role in modulating host immunity and preserving intestinal morphology, using an experimental infection model for *Salmonella* Typhimurium that had been previously standardized and validated in our laboratory under comparable experimental conditions (unpublished data).

## 2. Materials and Methods

### 2.1. Location, Birds, Treatment, and Growth Performance

The Institutional Animal Care and Use Committee of the University of Costa Rica approved all the animal experimentation methods utilized in this work (CICUA-9-2024). The trial was conducted at the climate-controlled housing experimental farm of the Avian Pathology Laboratory facilities of the School of Veterinary Medicine of the National University of Costa Rica, located in Heredia, Costa Rica (animal biosafety level 2/ABSL-2). The samples were analyzed at the Bacteriology and Avian Pathology Laboratory of the National University of Costa Rica and the Imunova Análises Biológicas LTDA, Brazil.

A total of 44 one-day-old Cobb 500 chicks were randomly allocated into four treatments, with 11 birds per cage (with shavings-covered bedding), during a 12-day experimental period. Treatments consisted of CT, control diet without a synbiotic, without any challenge; CT + Syn, control diet with a synbiotic, without any challenge; ST, control diet without a synbiotic, challenged with *S*. Typhimurium; ST + Syn, control diet with a synbiotic, challenged with *S*. Typhimurium. The study’s design aimed to validate the biological model, confirm the inducibility of the infectious process, and evaluate the efficacy of the synbiotic under challenge conditions. The CT group established physiological reference values; CT + Syn enabled the assessment of the baseline effects of supplementation; ST confirmed the reproducibility and effectiveness of the infection model; and ST + Syn allowed the determination of the protective and/or attenuating effects of the synbiotic against the infectious challenge.

The synbiotic product, PoultryStar^®^sol (dsm-firmenich, Vienna, Austria), was used in the drinking water during the first three days of life at a dose of 1 g/L of water, and PoultryStar^®^me (dsm-firmenich, Vienna, Austria) was used in the feed throughout the trial at a dose of 1 kg/ton of feed. Both products were composed of a prebiotic (fructooligosaccharide) and a probiotic mixture of five microbial strains selected from four different sections of the gastrointestinal tract in birds: *Lactobacillus reuteri* from the crop, *Enterococcus faecium* from the jejunum, *Bifidobacterium animalis* from the ileum, and both *Lactobacillus salivarius* and *Pediococcus acidilactici* were isolated from the cecum.

**Figure 1:**
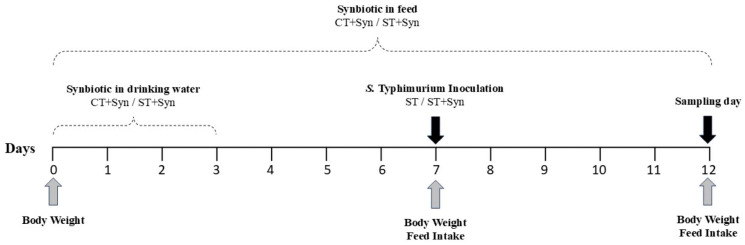
Outline of the experimental design indicating treatments, data collection, challenge, and sampling in broiler chicks.

An outline of the experimental design indicating the treatments and times of challenge is shown in Figure 1. The temperature and light were managed according to the broiler management guide [20]. Diets were formulated to meet the recommended level, as indicated by the genetic line [21]. This diet was non-medicated (without growth promoter, coccidia or mycotoxin treatment), vegetable-based (corn/soy), and in a mash form. All experimental diets were produced from this formulation in a single batch (i.e., the master control diet) to minimize ingredients and manufacturing variability among experimental treatments. After production of the master control diet, the feed was divided for each group, and the synbiotic was added to the corresponding treatments. On days 0, 7 and 12, the body weight (BW) was recorded.

### 2.2. Preparation of S. Typhimurium Inoculum and Oral Challenge

The strain *S*. Typhimurium ATCC 14028 of poultry origin was used to challenge the chickens. The strain was stored at −80 °C in the bacterial collection of the Bacteriology Laboratory of the School of Veterinary Medicine. The strain was thawed and inoculated onto Trypticase Soy Agar (TSA; Liofilchem™, Waltham, MA, USA) plates for 24 h at 36 ± 1 °C. After this incubation, two to three isolated colonies were selected and inoculated into a tube containing 10 mL of Trypticase Soy Broth (TSB; BD Difco™, Franklin Lakes, NJ, USA). The tube was then incubated for 24 h at 36 ± 1 °C. After incubation on the day of inoculation, the inoculum was diluted in Buffered Peptone Water (BPW; Neogen™, Lansing, MI, USA). The concentration of viable *S*. Typhimurium cells was estimated spectrophotometrically at 600 nm. After performing a 1:10 dilution of the original culture, an optical density (OD) of 0.290 was obtained, which corresponded to approximately 10^8^ CFU/mL according to Bergeron et al. [22]. Birds were challenged with 1 × 10^9^ CFU (verified by culture) per bird of the diluted inoculated broth orally by introducing the inoculum into the oral cavity using a sterile tuberculin syringe (BD Difco™, Franklin Lakes, NJ, USA) at 7 days of age [23,24,25].

### 2.3. Tissue and Sample Collection

On day 12 (5 days post infection, dpi), before each chick was euthanized, a blood sample was taken for intestinal permeability analysis. The birds were euthanized using a cervical dislocation technique. Necropsy was performed to collect samples for various analyses: histological evaluation of the duodenum, jejunum, ileum, cecum, liver, and heart; histomorphometric analysis of the ileum; immunofluorescence studies of the ileum and cecum; gene expression analysis of inflammatory mediators in the cecal tonsil; and additional samples of intestinal content and liver were collected for *Salmonella* culture and isolation. The intestinal sections were collected according to the protocol of Souza et al. [26].

### 2.4. Salmonella spp. Isolation and Identification from Intestinal and Hepatic Samples

Intestinal content and liver samples were collected in sterile sample bags and stored on ice before being delivered to the laboratory. A qualitative determination (presence/absence) of *Salmonella* spp., using the methods MLG 4.10 described by the USDA/FSIS, was performed [27]. Briefly, BPW was used as a pre-enrichment medium, with a 1:10 dilution of the sample (intestinal content and liver) added to the medium. The mixture was then incubated for 24 h at 36 ± 1 °C. Rappaport–Vassiliadis (Liofilchem™, Waltham, MA, USA) and Tetrathionate (Acumedia™, Lansing, MI, USA) were used as enrichment media; 1:100 (100 µL:10 mL) and 1:20 (500 µL:10 mL) dilutions were made of the sample in each medium, respectively, and incubated in a water bath at 42 ± 0.5 °C for 24 h. Xylose-Lysine-Tergitol 4 Agar (XLT4; Neogen™, Lansing, MI, USA) was used as the selective culture media. XLT4 samples were streaked and incubated at 35 ± 1 °C for 24 h. Typical *Salmonella* colonies (red colonies with a black center) were selected for analysis. Biochemical identification was performed using Triple Sugar-Iron Agar (TSI; BD Difco™, Franklin Lakes, NJ, USA), Lysine-Iron-Arginine Agar (LIA; Liofilchem™, Waltham, MA, USA), and Urea Agar (Acumedia™, Lansing, MI, USA). Strains exhibiting a TSI reaction of K/A with hydrogen sulfide production (H_2_S^+^), LIA reaction of K/NC, and a negative result on Urea Agar were presumptively identified as *Salmonella* spp. Confirmation of the suspected strains was carried out using the rapid plate agglutination test with omnivalent *Salmonella* antiserum (Mast™ Assure, *Salmonella* O, Mono Factor O4, Mast Group™, Merseyside, UK). The presence of visible agglutination on the plate was considered definitive confirmation. The outcomes of *Salmonella* isolation from the intestinal and hepatic samples were recorded as binary variables (positive or negative).

### 2.5. Immunofluorescence Detection of Salmonella spp. In Ileum and Cecum Tissue Sections

The immunofluorescence assay for *Salmonella* was performed according to the methods described by Insalata et al. [28] and Fantasia [29] with modifications. The ileum and cecum samples were collected (of 2 cm in size per bird) and immediately fixed in a zinc fixative. After 24 h in contact with the fixative, the sections were transferred to a new container containing alcohol (at 70%). The tissues were embedded in paraffin and sectioned into approximately 15 μm sections. Briefly, after deparaffinization and rehydration, the sections were incubated with primary polyvalent *Salmonella* antiserum A-E+Vi (Vircell™, Granada, Spain), followed by incubation with a goat anti-Rabbit IgG (H+L) Cross-Adsorbed Secondary Antibody (Alexa Fluor™ 488; Invitrogen™, Waltham, MA, USA). Sections were analyzed using an Olympus BX51 fluorescence microscope and analyzed using Cell^F software V2.8 (Olympus™, Tokyo, Japan). Images of the preparations were captured at 10× magnification in two different fields of view, and marker-positive cells were subsequently counted. Results were expressed as the average number of positive cells per sample (villus or area). Micrographs showing blue fluorescence represented DAPI staining of cell nuclei; green fluorescence, FITC, represented the *Salmonella* immunopositive reaction; and a blue and green fluorescence fusion.

### 2.6. Gene Expression by qPCR

The gene expression assay was performed according to Zhang et al. [30] with modifications. The primers used in this study were designed by Imunova Análises Biológicas LTDA, Brazil, from the *Gallus gallus* reference genome (accession GCA_016699485.1). Cecal tonsil segments were fixed in a molecular stabilizing solution (RNAlater™, Thermo Fischer Scientific™, San Jose, CA, USA). This analysis represented the final number of targets analyzed by the qPCR method as a relative gene expression value for the CT group. The selected targets were interleukin-1 beta (IL-1β); interleukin-2 (IL-2); interleukin-4 (IL-4); interleukin-6 (IL-6); interleukin-10 (IL-10); tumor necrosis factor alpha (TNF-α); interferon gamma (IFN-γ); transforming growth factor beta 1 (TGF-β1); and mucin 2 (MUC2). Approximately 100 mg of the cecal tonsil sample was mechanically homogenized, and the total RNA was extracted and purified using a commercial kit (MVXA-SU01 FAST™, Loccus™, Sao Paulo, Brazil). The extracts were treated with DNase, and the RNA was quantified and its purity assessed by spectrophotometry. After RNA extraction, cDNAs were synthesized using a cDNA synthesis kit, with 1 μg of RNA per reaction. Two normalizing genes were used as internal controls. The results were normalized by applying the protocol described by Livak and Schmittgen [31], using the method ΔΔCt (ΔCt/average of the control ΔCt), the CT group as a control.

### 2.7. Histopathology and Lesion Score

Samples of the heart, liver, duodenum, jejunum, ileum, cecum, and cecal tonsil were removed and then fixed with a 10% formaldehyde solution. The organ segments were dehydrated in an ascending ethanol gradient. These samples were then cleaned in xylene, embedded in paraffin wax, and sectioned into 5 to 8 µm thick sections. They were stained with hematoxylin and eosin (HE) and observed under an optical microscope [32]. A semi-quantitative analysis of the observed lesions was performed, detailing the characterization of the lesions according to the classification proposed by Prentza et al. [33] and Chen et al. [34]. Depending on the extent and severity, the lesions were classified as no injury (0), mild (1), moderate (2), severe (3), and very severe (4).

### 2.8. Ileum Histomorphometry

The ileum segments were subjected to histological processing and stained using the hematoxylin and eosin technique, as described above. The intestinal morphology of each sample was observed under a microscope (Discovery v12™, Zeiss™, Oberkochen, Germany), photographed with an Axiocam, and analyzed with Zen Blue Software V3.4 (Zeiss™, Oberkochen, Germany). Initially, a panoramic image of the entire section was captured, followed by three images of the tissue with a higher magnification. In each image, three different points were quantified. Using the methods described by Alshamy et al. [35], the villus height (VH), epithelial height (EH), crypt depth (CD), intestinal mucosal surface (IMS), muscular tunic thickness (MLT), and goblet cells (GC) were determined.

### 2.9. Intestinal Permeability

To evaluate intestinal permeability, fluorescein isothiocyanate dextran (FITC-d; 1.1 mg, 3–5 kDa, Sigma™, Sao Paulo, Brazil) was administered orally to a total of 44 chicks on day 12 (5 dpi). Two and a half hours after the gavage, approximately 2.0 mL of blood was collected by the femoral vein [36,37], and the birds were immediately euthanized by cervical dislocation. Following the procedure previously described by Vicuña et al. [38] and Liu et al. [36], the blood was centrifuged at 1000× *g* for 15 min, and 100 µL of the serum was used to calculate the FITC-d concentration. Serum fluorescence intensity was determined in a plate fluorometer (Varioska™, Thermo Fischer Scientific™, San Jose, CA, USA). A standard dilution curve of the reagent was used to determine the FITC-d concentration in the sample.

### 2.10. Statistical Analysis

The experiment was conducted using a completely random design applied at the level of bird assignment to each treatment group. The data were analyzed using Minitab statistical software version 22. Initially, the outlier identification test (ROUT, Q = 1) was performed. Subsequently, the data were subjected to the Shapiro–Wilk normality test, and based on this result, when the data followed a Gaussian distribution, they were analyzed using one-way ANOVA followed by Tukey’s multiple comparisons post-test. If the data did not follow a Gaussian distribution, they were analyzed using the Kruskal–Wallis test and Dunn’s multiple comparisons post hoc test. Data were expressed as the mean and pooled standard error of the mean (SEM), and *p* < 0.05 was considered statistically significant.

## 3. Results

### 3.1. Salmonella Recovery in the Intestine

**Figure 2:**
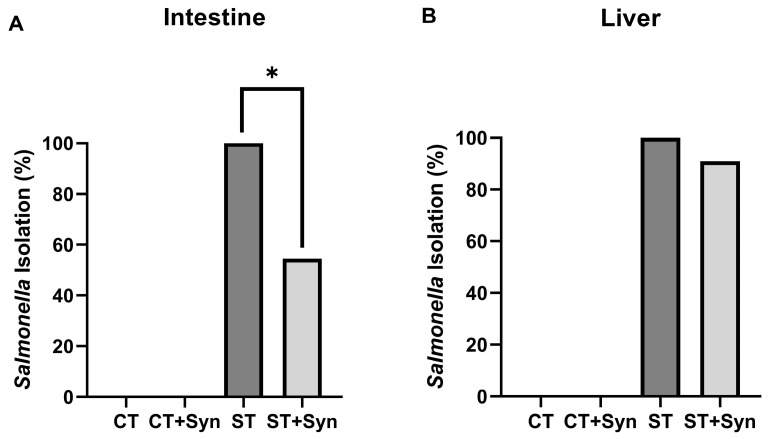
The effect of a synbiotic on the isolation of *S*. Typhimurium in the intestinal content (**A**) and liver (**B**). * Mean significant differences (*p*-value < 0.001). CT, control diet without a synbiotic, without any challenge; CT + Syn, control diet with a synbiotic, without any challenge; ST, control diet without a synbiotic, challenged with *Salmonella*; ST + Syn, control diet with a synbiotic, challenged with *Salmonella*. Data are expressed as the % *Salmonella* isolation (*n* = 11).

The effect of the synbiotic on the isolation of *Salmonella* from intestinal content and liver in groups challenged with *S*. Typhimurium is shown in Figure 2. In the ST group, a positive *Salmonella* culture was achieved in 100% of the intestine (Figure 2A) and liver (Figure 2B) samples. In contrast, in the ST + Syn group, only 54.4 and 90.9% were achieved in the intestine and liver, respectively (*p* < 0.01). All birds that received the *S*. Typhimurium inoculum were positive in at least one of the two sample types. Conversely, *Salmonella* was not isolated from any samples in the non-challenged groups.

### 3.2. Salmonella Colonization in the Ileum

**Figure 3:**
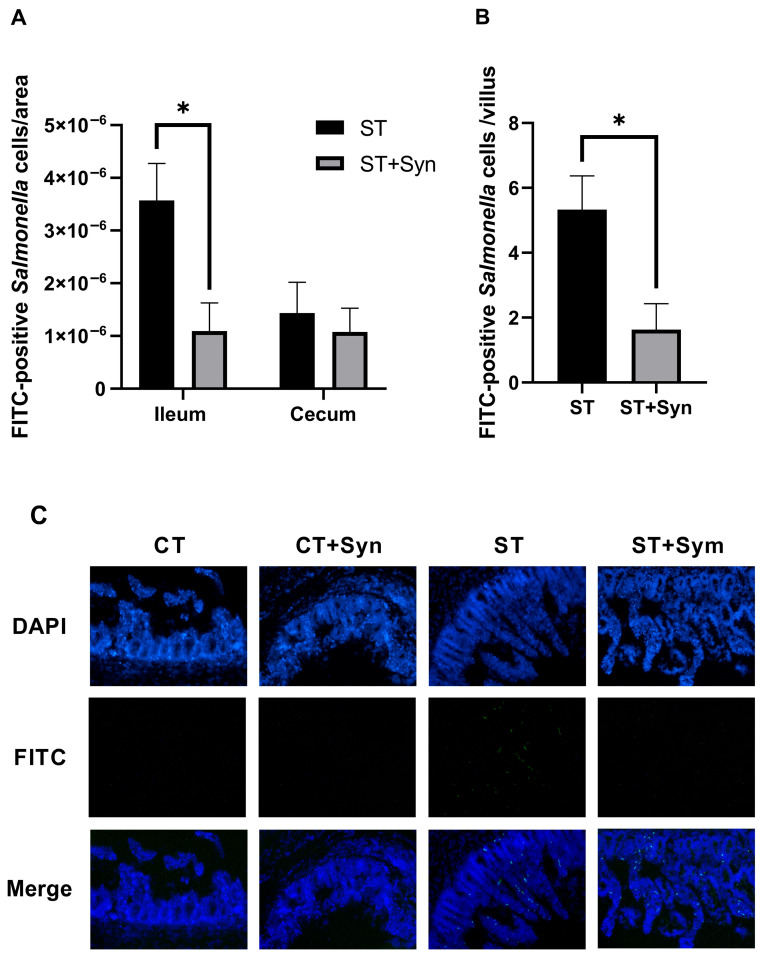
The effect of a synbiotic with and without *S*. Typhimurium challenge on bacteria adhesion to the ileum and cecum. * Mean significant differences (*p*-value < 0.05). (**A**) Bacterial densities in the ileum and cecum. (**B**) Number of infected cells per villus in the ileum. (**C**) Fluorescence microscopy showing bacterial adhesion to ileal and cecal tissue (DAPI staining for cell nuclei in blue, FITC staining for *Salmonella* in green). CT, control diet without a synbiotic, without any challenge; CT + Syn, control diet with a synbiotic, without any challenge; ST, control diet without a synbiotic, challenged with *Salmonella*; ST + Syn, control diet with a synbiotic, challenged with *Salmonella*. Data are expressed as means and pooled SEMs (*n* = 11).

The presence of *Salmonella* measured by immunofluorescence in the ileum and cecum of challenged broiler chickens is illustrated in Figure 3. Dietary supplementation with the synbiotic significantly decreased (*p* < 0.01) the density of bacteria per area (Figure 3A) and the number of infected cells per villus in the ileum (Figure 3B), while the density of bacteria per area in the cecum did not differ between treatments. All inoculated chickens showed a positive signal for *Salmonella* in the ileum and cecum. Fluorescence microscopy photographs showed bacterial adhesion to ileal and cecal tissue (Figure 3C, DAPI staining for cell nuclei in blue, FITC staining for *Salmonella* in green).

### 3.3. Inflammatory Response and Mucosal Protection

**Figure 4:**
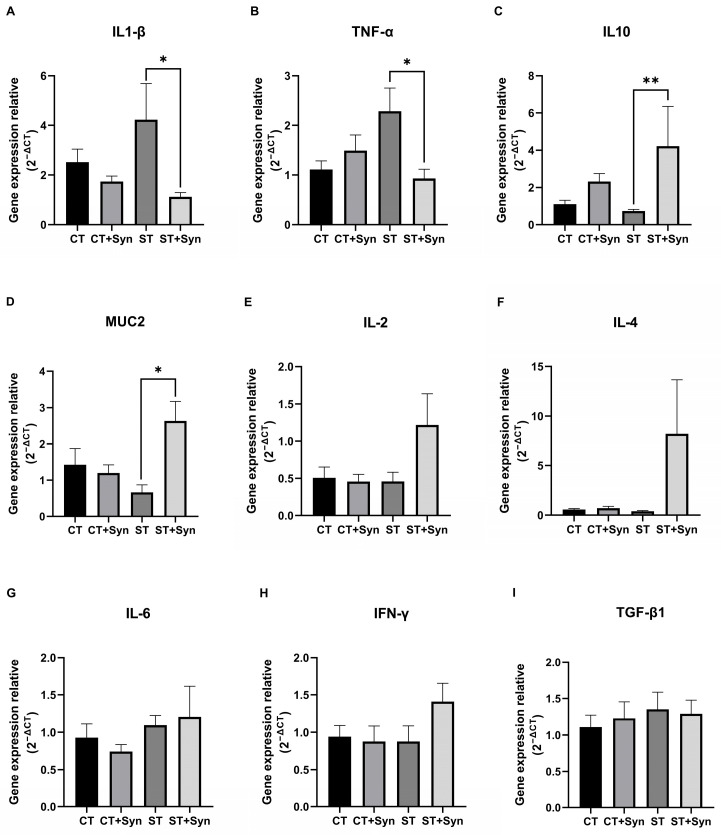
The effect of a synbiotic with and without *S.* Typhimurium challenge on the mRNA relative quantity of cytokines in the cecal tonsil in broiler chickens. (**A**) IL-1β. (**B**) TNF-α. (**C**) IL-10. (**D**) MUC2. (**E**) IL-2. (**F**) IL-4. (**G**) IL-6. (**H**) IFN-γ. (**I**) TGF-β1. * Mean significant differences (*p*-value < 0.05). ** Mean significant differences (*p*-value < 0.01). CT, control diet without a synbiotic, without any challenge; CT + Syn, control diet with a synbiotic, without any challenge; ST, control diet without a synbiotic, challenged with *Salmonella*; ST + Syn, control diet with a synbiotic, challenged with *Salmonella*. Data are expressed as means and pooled SEMs (*n* = 11).

At 5 dpi, synbiotic supplementation significantly affected the mRNA abundance of IL-1β (Figure 4A), TNF-α (Figure 4B), IL-10 (Figure 4C), and MUC2 (Figure 4D) in the cecal tonsils of *Salmonella*-challenged birds (*p* < 0.05). The challenged group receiving the synbiotic (ST + Syn) showed a higher relative expression of the anti-inflammatory cytokine IL-10 and a lower expression of the pro-inflammatory cytokines IL-1β and TNF-α compared with the non-supplemented challenged group, with values comparable to those observed in the non-challenged groups. In addition, MUC2 mRNA abundance was significantly higher in the ST + Syn group than in the ST group (*p* < 0.05), whereas no differences were detected among the non-challenged groups. Regarding IL-2, IL-4, IL-6, IFN-γ, and TGF-β1, these did not show statistically significant differences.

### 3.4. Tissue Damage and Inflammation

A pathological analysis of lesions based on the sections stained with hematoxylin and eosin showed that the group challenged with *S*. Typhimurium without supplementation (ST) exhibited the highest lesion scores. In contrast, the synbiotic-treated challenged group (ST + Syn) showed a significant reduction in lesion severity (Table 1; *p* < 0.05).

**Figure 5:**
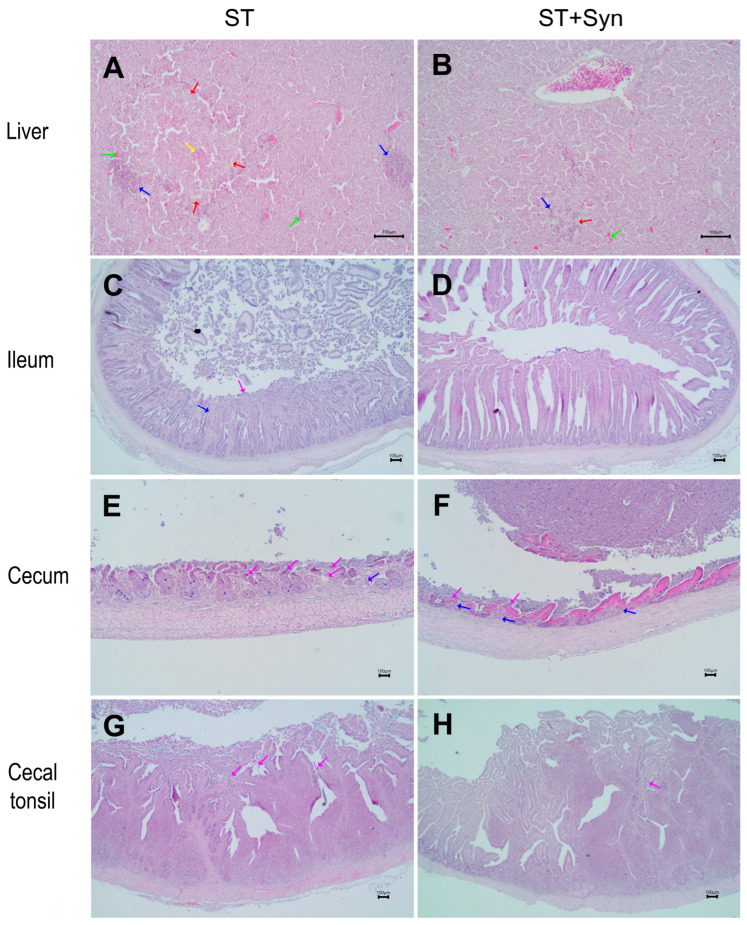
Morphologic alterations in the liver (**A**,**B**), ileum (**C**,**D**), cecum (**E**,**F**), and cecal tonsils (**G**,**H**) of broiler chickens infected with *S*. Typhimurium and treated with or without a synbiotic. Representative images of tissues stained with HE. ST, control diet without a synbiotic, challenged with *S*. Typhimurium (**A**,**C**,**E**,**G**); ST + Syn, control diet with a synbiotic, challenged with *S*. Typhimurium (**B**,**D**,**F**,**H**). Focal extensive area of necrosis (red arrow), hemorrhage (yellow arrow), and hyperemia (green arrow) with perivascular lymphoheterophilic infiltration in the liver of ST chickens (**A**) compared with a more restricted necrotic area in ST + Syn chickens (**B**). Villi tip enterocytes erosion and detachment in the ileum (magenta arrow) with edema and moderate inflammatory infiltration (blue arrow) in the lamina propria (**C**). Less severe lesions in ST + Syn chickens (**D**). Severe villi ulceration and detachment in the cecum of ST chickens (**E**). Atrophy and less inflammatory infiltration in the lamina propria of ST + Syn chickens, with extensive accumulation of detritus seen in the lumen (**F**). Slight enterocytes detachment in the tip of the villi in ST chickens (**G**). Less severe changes were observed in ST + Syn chickens (**H**).

Liver lesions indicated that the ST + Syn group showed a significant improvement in hepatic necrosis, edema, and congestion compared with the ST group (*p* < 0.05). No differences were observed between the non-challenged groups and the ST + Syn group. The challenged unsupplemented group presented more severe pathological changes, including disorganization of the hepatic cords, sinusoidal dilation with congestion, edema, Kupffer cell hyperplasia, and focal necrotic areas surrounded by marked inflammatory cell infiltration composed of heterophils and macrophages (Figure 5A). In contrast, the challenged supplemented group exhibited pronounced protective effects, with a nearly normal hepatic architecture comparable to that of the non-challenged groups (Figure 5B).

Histopathological evaluation revealed lesions in the duodenum, jejunum, and ileum. No significant differences were observed among the CT, CT + Syn, and ST + Syn groups. However, when compared with these groups, the ST group exhibited more severe heterophilic and mononuclear inflammatory infiltration, as well as vascular alterations including congestion, hemorrhage, and cellular necrosis (*p* < 0.05; Figure 5C). In the ileum, the ST + Syn group showed a higher degree of enterocyte vacuolar degeneration compared to the other treatments (*p* < 0.05; Figure 5D).

Assessment of histopathological lesions in the cecum demonstrated a significant difference in injury severity between the ST and ST + Syn groups. The ST + Syn group exhibited reduced inflammatory infiltrate and mucosal ulceration compared with the ST group (*p* < 0.05; Figure 5E,F). Lesions in the cecal tonsil indicated that synbiotic supplementation decreased the severity of necrosis at the villus tip and within the lamina propria, as well as inflammatory infiltration and vascular alterations (*p* < 0.05; Figure 5G,H). Conversely, increased lymphoid tissue proliferation and enterocyte vacuolar degeneration were observed in the ST + Syn group (*p* < 0.05). No significant differences were detected between the non-challenged groups and the ST + Syn group.

### 3.5. Ileal Epithelial Morphometry

The effect of the synbiotic on histomorphometry characteristics of the ileum is represented in Table 2. Supplementation with the synbiotic significantly affected epithelial height; the ST + Syn group had a greater height than the ST group, with no difference observed between the ST + Syn group and those not challenged with the bacteria (*p* < 0.05).

### 3.6. Intestinal Permeability

**Figure 6:**
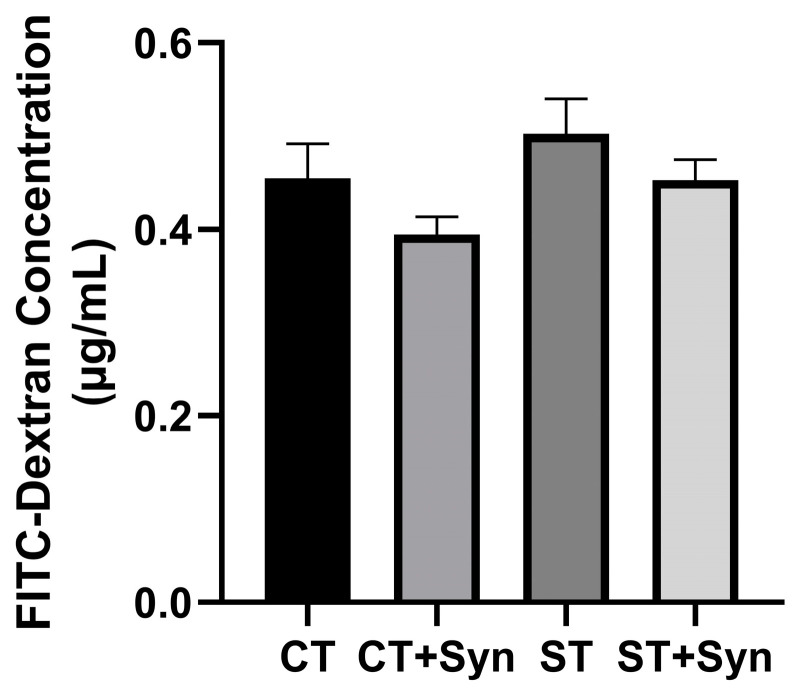
The effect of a synbiotic with and without *S*. Typhimurium challenge on gut permeability, as tested at 5 dpi. CT, control diet without a synbiotic, without any challenge; CT + Syn, control diet with a synbiotic, without any challenge; ST, control diet without a synbiotic, challenged with *Salmonella*; ST + Syn, control diet with a synbiotic, challenged with *Salmonella*. Data are expressed as means and pooled SEMs (*n* = 11).

There were no significant differences between the groups as shown in Figure 6, as exposure to *S*. Typhimurium did not result in an increase in intestinal permeability (measured with FITC-dextran) 5 days after exposure when compared to unexposed birds.

### 3.7. Body Weight

The effects of the synbiotic with and without *S*. Typhimurium challenge on the body weight (BW) of broiler chickens are shown in Table 3. On day 7, prior to the *Salmonella* challenge, the BW was higher in the CT + Syn and ST + Syn groups that received the synbiotic treatment compared to the groups that were not treated (*p* < 0.05). On day 12 (5 dpi), the groups challenged with *Salmonella* presented a lower BW compared to the non-challenged groups. The ST + Syn group presented a higher BW compared to the other group challenged without the synbiotic (ST, *p* < 0.05).

## 4. Discussion

Our results showed a positive immune response in broiler chickens and the early protective effect of a synbiotic using a reduced in vivo experimental model of *S*. Typhimurium infection. The protective effect appeared to be sufficient to attenuate an infection and its consequences with a high inoculum concentration (1 × 10^9^ CFU), as used in our trial, although some research [39,40] has indicated that environmental exposure of chicks to *Salmonella* sp. during the first days of life can probably be limited to low to medium concentrations.

In our study, the isolation of *S*. Typhimurium in the gut decreased significantly at 5 dpi when supplemented with the synbiotic. This finding was consistent with other studies which have shown that supplementation can reduce *Salmonella* colonization patterns [12,41,42,43]. However, the results were variable, and the differences could be attributed to variations with in vivo models [12], differences in the breed of birds used for the challenge [44], and the virulence of the specific *Salmonella* strains used [45], the latter of which could be crucial due to the differences in adhesion and invasion capacity between serovars. *Salmonella* recovery in internal organs can describe the translocation of bacteria; in this case, from the gut to the liver. This bacterial translocation can be used as an indicator of intestinal health to describe the alteration of the epithelial lining of the gastrointestinal tract [24,43].

For bacterial pathogenesis during *Salmonella* infection, successful colonization is crucial [46]. This is a key aspect of its invasion mechanism and virulence factors that ensure its survival and reproduction [47]; colonization levels can directly affect infection severity [34]. It appeared that supplementation with the synbiotic became relevant at this point. Our results showed that supplementation was able to reduce the density of bacteria and the number of infected cells per villus in the ileum, which was consistent with other reports [46,48], where the use of prebiotics/probiotics caused a decrease in the *Salmonella* load in the intestine. The probiotic bacteria contained in the synbiotic have been shown to successfully colonize the chicken intestine, in addition to producing enterocins, pediocins, and lactic acid that can inhibit the growth [48] and prevent the adhesion of enteric pathogens in the intestine [49]. The results showed no differences between treatments in the cecum, which may be due to the organ’s critical role in *Salmonella* colonization, as its slow intestinal content flow and fermentation processes can create favorable conditions for bacterial persistence [25]. Some studies established that the cecum above the ileum was the preferred location in the gastrointestinal tract for colonization by *S*. Typhimurium [50] and *S.* Enteritidis [51], which could explain why the protective scope of the synbiotic was not reflected in this organ; in contrast, significant reductions in the amount of *Salmonella* in the cecum of chicks treated with probiotics were reported [52].

In our study, synbiotic supplementation decreased the adhesion and invasion capacity of *S*. Typhimurium to the ileum epithelium, explaining the difference in positive results between culture and immunofluorescence in the intestine. It is possible that the reduction in the *Salmonella* load achieved was sufficient to prevent recovery during laboratory isolation. Furthermore, the lower abundance of *S*. Typhimurium in the supplemented groups may contribute to a lower amount of LPS in the gastrointestinal tract, resulting in a reduced subsequent inflammatory response [9].

In the present infection study, analysis of the immune response, as measured by gene expression of inflammatory mediators, revealed an altered cytokine response in infected birds compared to uninfected controls, confirming the success of the infection. The exposure of the birds to a high dose of the pathogen likely triggered a more acute immune response and overexpression of immune-related genes, compared to the absence of or lower challenge doses [40]; in contrast, it should also be considered that supplementation with the synbiotic in *Salmonella*-infected birds decreased the pathogen load, and this was also likely to decrease the mRNA expression of cytokines [48,53].

The synbiotic treatment affected the gene expression of four of the eight genes investigated. The change in gene expression, whether positive or negative, affected or harmed epithelial integrity [43], which may explain why the immune system played a role in controlling *Salmonella* sp. translocation [25] and promoting immune tolerance [54]. Other researchers have reported that birds treated with probiotics/prebiotics and challenged with *S*. Typhimurium [9], *S*. Enteritidis [48], and *S*. Pullorum [34] showed suppression in the gene expression of inflammation-related cytokines. Similarly, gene expressions of anti-inflammatory cytokines were simultaneously promoted in this experiment. Although inflammatory cytokine production is necessary to elicit an active and protective response to a challenge, an excessive inflammatory response can unfortunately cause tissue damage [55]. In fact, the overexpression of pro-inflammatory cytokines can be the primary biomarker of an exacerbated intestinal inflammatory response [47].

The mechanism of supplementation in alleviating the inflammatory effects of *Salmonella* infection may be attributed to the promotion of increased expression of anti-inflammatory cytokines [9,55]. The expression of the pro-inflammatory cytokines IL-1 and TNF was related to an acute immune response against *Salmonella* sp. in young chicks [16,47] and induced the redistribution of tight junction proteins [56]. An elevated IL-10 expression can downregulate the production of pro-inflammatory cytokines by antigen-presenting cells and T lymphocytes. Concurrently, interferons (IFNs) supported epithelial regeneration and stimulated dendritic cells to induce IL-10-producing cell populations [53,57]. These cytokine profiles were associated with a tolerance response [58] and immune resistance to *Salmonella* in chickens [16].

In our study, the challenged and synbiotic-supplemented groups of birds showed increased expression of mucin 2 mRNA abundance compared to the other challenged birds that were not treated. Mucin 2 (MUC2) is a crucial component of the mucus layer and the primary line of defense for protecting epithelial cells in the intestinal tract from pathogens [55]. An increased MUC2 gene expression can lead to an enhanced barrier function of the epithelial lining, thereby protecting against the translocation of bacteria from the intestinal lumen into the bloodstream [43]. These results were consistent with other reports on synbiotic supplementation [43,55], which concluded that mucin 2 was essential for maintaining intestinal mucosa integrity. In contrast, other studies have found no differences in the MUC2 gene expression during exposure to *Salmonella* [25,59]. One of the main reasons for these differences was the time elapsed from infection to sampling, as it has been indicated that chickens tended to recover after 20 dpi [25]. Infection with *S*. Typhimurium and other enteropathogens significantly reduced intestinal mucin as a pathophysiological strategy [46], suggesting that measuring MUC2 gene expression may be a potential biomarker of intestinal health [43].

Descriptive histopathological examinations of sections from different parts of the intestine, liver, and heart from all the groups revealed a certain degree of infiltration by inflammatory cells, with a predominance of mononuclear cells and heterophils, indicating a basal level of inflammation. Commonly referred to as sterile inflammation, it is a chronic, low-grade process caused by non-infectious stimuli related to chemical, physical, and metabolic agents [60], which was further compounded by the fact that the feed offered to the birds did not contain any prophylactic treatment. More severe damage was observed in infected animals that were not supplemented with the synbiotic. This type of infiltration in the intestinal epithelium of chickens infected with *Salmonella* was considered a classic sign of an inflammatory immune response to the pathogen. With it, an increase in the expression of pro-inflammatory cytokines would be expected, accompanied by a reduction in the expression of the anti-inflammatory cytokine [61], which was also observed in our trial for untreated infected birds. Other reports concurred with our findings [9,34,53], suggesting that supplementation reduced inflammatory cell infiltration and maintained the integrity of intestinal epithelial cells. This result was attributed to the fact that the probiotic component enhanced the colonization of beneficial bacteria in the intestine, thereby reducing inflammation and increasing mucus production, which in turn strengthened the intestinal barrier [54].

Furthermore, HE staining revealed vacuolar degeneration of the enteric epithelium in the ileum and cecal tonsil of the challenged and supplemented group, indicating an active response to the specific *S*. Typhimurium infection [46]. Additionally, this same group reported a greater proliferation of lymphoid tissue in the cecal tonsil, which was attributed to the direct interaction of the synbiotic bacteria with the gut-associated lymphoid tissue (GALT). Binding probiotic bacteria to lymphocyte receptors activated immune cells, leading to their proliferation and an enhanced immune response [62]. This knowledge can provide insight into the function and intensity of immune cell responses following *Salmonella* infection and will contribute to the development of immunomodulatory strategies [51].

As inflammatory damage and vascular alterations were observed, several morphological changes can occur when a pathogen crosses the mucosal layer and adheres to the epithelium. The morphology of these components can be a key indicator of overall intestinal health [61]. *Salmonella* infection generally causes an increase in the cell turnover rate in the host intestine in response to infection [25], which can reduce the absorptive surface area due to epithelial exfoliation [49], goblet cell exfoliation [61], villous shedding [46], and excessive proliferation of intestinal crypt cells [50]. Our trial only showed a change in epithelial height. Evidence on the use of synbiotics in the literature related to the protective factor may vary due to the variety of probiotic strain formulations that they consist of, the pattern and age of infection, the number of days after infection at the time of sample collection, and the bacterial strain used for the challenge [33,59]. However, a protective effect has been reported by reducing crypt depth [49], lengthening villi [4,33,63], and stimulating goblet cell proliferation [49,55].

According to our results, neither exposure to *S*. Typhimurium nor synbiotic supplementation showed a change in the levels of FITC-d quantification in the serum of individuals exposed at 5 dpi. *Salmonella* infection can significantly inhibit the expression of claudins, occludins, cadherins, and MUC2 genes [34,59], indicating that the bacteria may disrupt the formation of tight junctions between intestinal epithelial cells [47,64]. Some studies have indicated that intestinal permeability was affected 72 h after exposure to *Salmonella* infection [65]; however, no differences were reported in assays that performed infections and measured at 6 dpi [25] and 7 dpi [59], while others have reported evidence of intestinal leakiness up to 10 dpi [64] and 14 dpi [24]. Differences in intestinal permeability could be influenced by the dose, age of exposure, feeding strategy, and virulence of the strain used [25], and this could also be related to intestinal immune regulation that was conditioned at the time of infection, as well as the maturation of the immune system [40].

The results of the trial indicated that the growth performance of the group exposed to *S*. Typhimurium infection and supplemented with the synbiotic was similar to that of the unexposed groups and significantly better than the other infected group, indicating that the treatment was successful in preventing aspects of growth depression associated with a direct challenge from the pathogen. Early inoculation with probiotic bacteria and prebiotics may allow for improved nutrient absorption and the expression of intestinal protective factors. The improved weight gain in supplemented birds has been attributed to the development of a gastrointestinal environment that favors enzymatic digestion and preserves the integrity of the intestinal epithelium [11]. Supplementation improved the absorption of proteins, minerals, vitamins, and amino acids, while modulating the composition and activity of the gut microbiota, thereby promoting muscle development and increasing protein content [53,66]. Similar results have been reported with the same synbiotic in trials with infectious challenges involving *Salmonella* [12,42,48], *Eimeria* spp. [54,67,68], *E. coli* [69,70], *C. perfringens* [53,54], and *C. jejuni* [71]. In contrast, studies performed by Cason et al. [72] and Shah et al. [73] described that the supplement did not influence body weight gain during the evaluation period.

## 5. Conclusions

Synbiotic supplementation demonstrated a protective effect against early experimental infection with *Salmonella* Typhimurium in broiler chickens. This protective action was evidenced by a reduction in bacterial colonization and epithelial adhesion, along with a favorable modulation of the host immune response and preservation of intestinal morphology integrity. Furthermore, these effects were associated with sustained body growth during the early stages of infection, as evidenced by a higher body weight compared with the challenged birds that did not receive the supplementation. This finding suggests that the intervention mitigated the negative impact of the infectious challenge, even under the controlled conditions of a reduced in vivo experimental model. Overall, these findings support the protective role of synbiotics previously reported in the literature and can substantiate their mechanism of action as an effective nutritional strategy to enhance intestinal health and disease resistance in broiler chickens

## Figures and Tables

**Table 1:** Histopathological lesion scoring of the heart, liver, and different sections of the intestine in chickens with and without exposure to *S.* Typhimurium.

Organ	Lesion	Lesion Extent and Severity				SEM	*p*-Value
		CT	CT + Syn	ST	ST + Syn		
Heart	Cardiomyocyte necrosis	0.00 ^b^	0.09 ^b^	0.81 ^a^	0.27 ^b^	0.46	0.001
	Lymphocytic infiltrate	0.09 ^b^	0.00 ^b^	0.45 ^a^	0.00 ^b^	0.37	0.020
Liver	Hepatocyte necrosis	0.00 ^b^	0.00 ^b^	1.54 ^a^	0.36 ^b^	0.76	0.004
	Edema	0.00 ^b^	0.00 ^b^	0.45 ^a^	0.00 ^b^	0.34	0.006
	Congestion	0.09 ^b^	0.09 ^b^	0.54 ^a^	0.09 ^b^	0.33	0.015
Duodenum	Heterophilic infiltrate	0.00 ^b^	0.00 ^b^	0.45 ^a^	0.00 ^b^	0.34	0.006
	Congestion	0.00 ^b^	0.09 ^b^	0.36 ^a^	0.00 ^b^	0.29	0.018
Jejunum	Crypt cell necrosis	0.00 ^b^	0.00 ^b^	0.63 ^a^	0.09 ^b^	0.43	0.031
	Congestion	0.09 ^b^	0.09 ^b^	0.81 ^a^	0.18 ^b^	0.52	0.005
Ileum	Degeneration of mucosal epithelial cells	0.00 ^b^	0.00 ^b^	0.00 ^b^	0.36 ^a^	0.25	0.002
	Enterocyte necrosis	0.27 ^b^	0.09 ^b^	1.00 ^a^	0.36 ^b^	0.49	0.001
	Hemorrhage	0.00 ^b^	0.09 ^b^	0.45 ^a^	0.00 ^b^	0.30	0.028
	Mononuclear infiltrate	0.90 ^b^	0.72 ^b^	1.63 ^a^	0.81 ^b^	0.48	0.025
	Heterophilic infiltrate	0.81 ^b^	0.63 ^b^	1.63 ^a^	0.81 ^b^	0.51	0.003
	Congestion	0.00 ^b^	0.09 ^b^	0.54 ^a^	0.00 ^b^	0.30	0.022
Cecum	Heterophilic infiltrate	1.18 ^b^	1.27 ^b^	2.36 ^a^	1.27 ^b^	0.81	0.004
	Mononuclear infiltrate	1.27 ^b^	1.18 ^b^	2.27 ^a^	1.27 ^b^	0.84	0.012
	Mucosal ulceration	0.00 ^b^	0.00 ^b^	0.72 ^a^	0.09 ^b^	0.48	0.002
Cecal tonsil	Degeneration of mucosal epithelial cells	0.00 ^b^	0.00 ^b^	0.00 ^b^	0.52 ^a^	0.26	0.001
	Proliferation of lymphoid tissue	0.00 ^b^	0.00 ^b^	0.00 ^b^	0.36 ^a^	0.25	0.002
	Mononuclear infiltrate	1.00 ^b^	0.72 ^b^	1.63 ^a^	0.90 ^b^	0.49	0.001
	Heterophilic infiltrate	1.00 ^b^	0.81 ^b^	1.63 ^a^	0.81 ^b^	0.54	0.003
	Villus tip necrosis	0.36 ^b^	0.36 ^b^	1.18 ^a^	0.36 ^b^	0.66	0.011
	Hemorrhage	0.09 ^b^	0.09 ^b^	0.72 ^a^	0.09 ^b^	0.41	0.001
	Lamina propria necrosis	0.00 ^b^	0.00 ^b^	0.72 ^a^	0.00 ^b^	0.59	0.013
	Congestion	0.00 ^b^	0.18 ^b^	0.63 ^a^	0.00 ^b^	0.39	0.001

^a,b^ Means in the same line with different superscripts are statistically different (*p*-value < 0.05). Depending on the extent and severity, the lesions were classified as no injury (0), mild (1), moderate (2), severe (3), and very severe (4). CT, control diet without a synbiotic, without any challenge; CT + Syn, control diet with a synbiotic, without any challenge; ST, control diet without a synbiotic, challenged with *Salmonella*; ST + Syn, control diet with a synbiotic, challenged with *Salmonella*. Data are expressed as means and pooled SEMs (*n* = 11).

**Table 2:** Effect of synbiotic with and without *S.* Typhimurium challenge on histomorphometry characteristics of the ileum in chickens.

Treatment	VH (µm)	EH (µm)	CD (µm)	IMS (µm)	MLT (µm)	GC (Cell/Villus)	VH:CD
CT	397.70	20.44 ^a^	57.71	47,382.49	244.50	73.91	6.61
CT + Syn	418.10	20.53 ^a^	54.66	63,960.95	291.50	77.29	7.24
ST	359.10	19.48 ^b^	60.63	38,100.96	236.60	70.38	6.50
ST + Syn	415.60	21.56 ^a^	57.34	47,959.39	290.90	73.05	7.21
SEM	74.50	1.29	5.98	22,087.10	66.45	11.79	1.17
*p*-value	0.308	0.013	0.192	0.059	0.138	0.629	0.377

^a,b^ Means in the same column with different superscripts are statistically different (*p*-value < 0.05). CT, control diet without a synbiotic, without any challenge; CT + Syn, control diet with a synbiotic, without any challenge; ST, control diet without a synbiotic, challenged with *Salmonella*; ST + Syn, control diet with a synbiotic, challenged with *Salmonella.* VH, villus height; EH, epithelial height; CD, crypt depth; IMS, intestinal mucosal surface; MLT, muscular layer thickness; GC, goblet cells. Data are expressed as means and pooled SEMs (*n* = 11).

**Table 3:** The effect of a synbiotic with and without *S*. Typhimurium challenge on the body weight.

Treatment	Day 1	Day 7	Day 12
CT	40.820	111.545 ^b^	259.100 ^bc^
CT + Syn	39.364	128.455 ^a^	290.545 ^a^
ST	40.273	112.182 ^b^	251.455 ^c^
ST + Syn	40.090	127.364 ^a^	279.091 ^ab^
SEM	3.454	13.166	22.774
*p*-value	0.802	0.031	0.018

^a,b,c^ Means in the same column with different superscripts are statistically different (*p*-value < 0.05). CT, control diet without a synbiotic, without any challenge; CT + Syn, control diet with a synbiotic, without any challenge; ST, control diet without a synbiotic, challenged with *Salmonella*; ST + Syn, control diet with a synbiotic, challenged with *Salmonella*. Data are expressed as means and pooled SEMs (*n* = 11).

## Data Availability

The original contributions presented in this study are included in the article. Further inquiries can be directed to the corresponding author.
